# Predictors of mortality in chronic obstructive pulmonary disease: a systematic review and meta-analysis

**DOI:** 10.1186/s12890-022-01911-5

**Published:** 2022-04-04

**Authors:** Catherine Owusuaa, Simone A. Dijkland, Daan Nieboer, Carin C. D. van der Rijt, Agnes van der Heide

**Affiliations:** 1 Department of Medical Oncology, Erasmus MC Cancer Institute, PO Box 2040, 3000 CA Rotterdam, The Netherlands; 2 Department of Public Health, Erasmus MC, Erasmus University Medical Center, PO Box 2040, 3000 CA, Rotterdam, The Netherlands

**Keywords:** Advance care planning, Predictors, Mortality, Chronic obstructive pulmonary disease

## Abstract

**Background:**

Better insight in patients’ prognosis can help physicians to timely initiate advance care planning (ACP) discussions with patients with chronic obstructive pulmonary disease (COPD). We aimed to identify predictors of mortality.

**Methods:**

We systematically searched databases Embase, PubMed, MEDLINE, Web of Science, and Cochrane Central in April 2020. Papers reporting on predictors or prognostic models for mortality at 3 months and up to 24 months were assessed on risk-of-bias. We performed a meta-analysis with a fixed or random-effects model, and evaluated the discriminative ability of multivariable prognostic models.

**Results:**

We included 42 studies (49–418,251 patients); 18 studies were included in the meta-analysis. Significant predictors of mortality within 3–24 months in the random-effects model were: previous hospitalization for acute exacerbation (hazard ratio [HR] 1.97; 95% confidence interval [CI] 1.32–2.95), hospital readmission within 30 days (HR 5.01; 95% CI 2.16–11.63), cardiovascular comorbidity (HR 1.89; 95% CI 1.25–2.87), age (HR 1.48; 95% CI 1.38–1.59), male sex (HR 1.68; 95% CI 1.38–1.59), and long-term oxygen therapy (HR 1.74; 95% CI 1.10–2.73). Nineteen previously developed multicomponent prognostic models, as examined in 11 studies, mostly had moderate discriminate ability.

**Conclusion:**

Identified predictors of mortality may aid physicians in selecting COPD patients who may benefit from ACP. However, better discriminative ability of prognostic models or development of a new prognostic model is needed for further large-scale implementation.

*Registration*: PROSPERO (CRD42016038494), https://www.crd.york.ac.uk/prospero/.

**Supplementary Information:**

The online version contains supplementary material available at 10.1186/s12890-022-01911-5.

## Key message

- This article describes a systematic review that describes the predictors of mortality in patients with chronic obstructive pulmonary disease. The results indicate that the previous hospitalizations for acute exacerbation, cardiovascular mortality, male sex, long-term oxygen therapy, and other multicomponent prognostic models could aid physicians in timely advance care planning.
- Multiple predictors of mortality can aid physicians in timely advance care planning in patients with chronic obstructive pulmonary disease. However, combining these predictors in prognostic models with adequate performance requires more research.

## Background

Advance care planning (ACP) allows patients and their physicians to make plans for future healthcare [1]. In ACP, patients’ goals and preferences regarding future medical treatment and care are discussed. ACP is aimed at improving the quality of care for chronically ill patients, especially those who are nearing their end of life. This also holds for patients with advanced stages of chronic obstructive pulmonary disease (COPD), which is the third leading cause of mortality globally [2].

ACP discussions are not yet standard practice for patients with COPD [3]. Physicians seem to find it important to identify the patients who qualify for such discussions, but they are often uncertain about when to start them [4]. This uncertainty may be due to the gradual functional decline that patients with COPD typically experience [5]. That gradual decline is often interrupted by acute exacerbations, which may not only lead to hospitalization but are also associated with an increased in-hospital mortality risk [6]. Mortality rates ranged from 1.8 to 20.4% within three months after a hospitalization, and from 18.8 to 45.4% for the period from three to 24 months after a hospitalization [7].

Internationally, several quality frameworks strongly advise the initiation of ACP for patients in the last year of life, although patients’ preferences may differ regarding the start of ACP. Advance care planning is probably most efficient when started at least several months before the patient dies. Better and timely insight in patients’ mortality risk may thus support physicians in the timely initiation of ACP discussions. Several predictors of mortality have been studied for COPD, such as age, gender, body mass index, comorbidity, and forced expiratory volume in one second (FEV_1_) [7]. But consensus on its use in multivariable prognostic model has not been reached. We aimed to provide an overview of predictors and prognostic models for mortality within 3–24 for patients with COPD. Furthermore, we studied the discriminative ability of the multicomponent prognostic models for mortality.

## Methods

### Search strategy and selection process

The study protocol for this review and meta-analysis (also including a search for advanced cancer) on predictors of mortality in patients with COPD was published on PROSPERO with registration number CRD42016038494; link https://www.crd.york.ac.uk/PROSPERO/display_record.php?RecordID=38494. One researcher (CO) and an information specialist from the Erasmus MC Medical Library developed the search strategy, which consisted of the terms “obstructive airway disease”, “COPD”, “prediction”, and “mortality”. They performed the search in the following databases in April 2020: Embase, PubMed, MEDLINE, Web of Science, and Cochrane Central (Additional file 1: e-Table S1). All identified papers were downloaded into a reference management program.

Studies reporting on single predictors as well as studies reporting on prognostic models were eligible. Both observational and experimental studies were included if they: studied patients with COPD; studied mortality within a follow-up period of 3–24 months; presented risk estimates (hazard ratio, odds ratio, or relative risk) with corresponding standard errors of predictors or presented the performance (model discrimination: the ability to distinguish survivors and non-survivors, measured with the c-statistic or area under the receiver operating characteristic curve [AUC]; and model calibration: observed versus predicted risk of mortality) of prognostic models; and were published in English in the year 2000 or later. We excluded studies that examined predictors outside the follow-up period of 3–24 months and systematic reviews and meta-analyses.

CO initially screened the titles of all papers, followed by independent screening of the abstracts by CO and SAD. The decision to include a study was based on full texts, which were requested from its first author if unavailable. Disagreements regarding inclusion of studies were solved by discussion or by involvement of two other researchers (CCDR and AH). The researchers also hand-searched the reference lists of included studies and of systematic reviews to include other relevant studies.

### Data extraction

CO and SAD independently extracted information from each included study on first author, publication year, design, population (sample size, age, sex, average FEV_1_), follow-up period, mortality rate, risk estimates and standard errors, and if available, the discriminative ability of a prognostic model. All means or medians were derived with standard deviations or interquartile range, respectively. In case different studies examined the same study data, we selected the most recent publication. In addition, when studies reporting estimates of mortality risk covered different follow-up periods, we selected the longest follow-up.

Two researchers independently scored all studies on risk-of-bias with a customized appraisal checklist that consisted of the Quality in Prognosis Studies tool, complemented with items from the Critical Appraisal and Data Extraction for Systematic Reviews of Prediction Modelling Studies checklist (Additional file 1: e-Table S2) [8, 9]. Studies were scored as low, moderate, or high risk-of-bias based on six domains: study participation, study attrition, predictors, outcome, statistical analysis, confounding, and, if applicable, prognostic model performance. The researchers resolved any disagreements in the risk-of-bias scoring through discussion. This study was reported according to the Preferred Reporting Items for Systematic and Meta-analyses (PRISMA) guideline [10].

### Data analysis

A meta-analysis was performed, in which we pooled predictors with the same measuring units or cut-offs, from multivariable analyses [11]. We calculated the overall risk estimate and standard error in a fixed-effects model for predictors pooled from two studies, and a random-effects model for predictors pooled from three or more studies. For predictors in a random-effects model, we calculated the between-study heterogeneity with the *I*^2^*-*statistic [12]. A heterogeneity of 0–30% was considered as insignificant, 40–60% as moderate, and > 60% as substantial. We examined the possibility to perform an additional meta-analysis with low risk-of-bias studies only. All meta-analyses were performed using Cochrane’s Review Manager Version 5.3.

Furthermore, we summarized the variables and discriminative ability of multivariable prognostic models. Discriminative ability is generally expressed in terms of the AUC or c-statistic, ranging from 0.5 (no discrimination) to 1 (perfect discrimination) [13]. Funnel plots were used to detect publication bias [14].

## Results

**Fig. 1:**
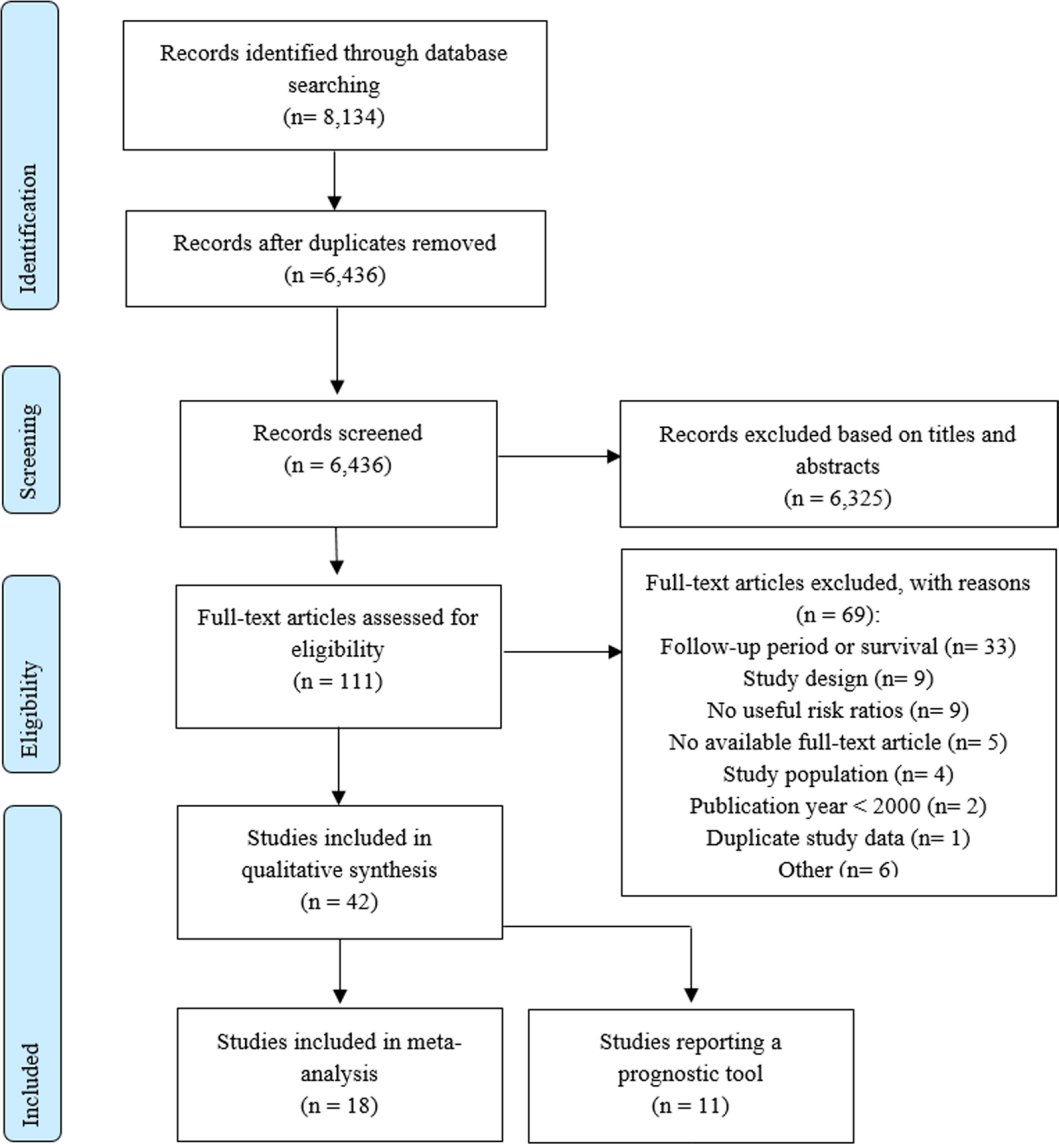
Study selection

Our search resulted in 6,436 studies after removing duplicates, of which we excluded 6,325 after screening titles and abstracts (Fig. 1). Based on the full texts of the remaining 111 studies, we included 42 studies for a qualitative synthesis, of which 18 studies were included in the meta-analysis for different predictors and eleven studies reported on multicomponent prognostic models. Forty studies were observational cohort studies and two studies were secondary analyses of randomized controlled trials. The studies had sample sizes ranging from 49–418,251 patients’ mean or median age ranging from 53.0–81.7 years, follow-up periods ranging from 6–24 months, and mortality rates ranging from 5.1–65.0% (Table 1). Six studies were scored as having a low risk-of-bias, 28 as having a moderate risk-of-bias, and eight as having a high risk-of-bias (Table 2).

**Table 1:** Study details and patient characteristics

References	Design	N	Study population/average FEV_1_ (% predicted)	Age	Men, %	Follow-up, months	Mortality rate, %	Meta-analysis (N = 17)	Model
Abu Hussein [21]	Cohort	646	COPD, GOLD I-IV/FEV_1_ 52.4 ± 26.0	67.4 ± 10.3	61.6	24	8.1	−	−
Almagro [22]	RCT	606	COPD exacerbation/FEV_1_ 45.4 ± 15.6 (cohort 1) & 41.2 ± 14.8 (cohort 2)	72.6 ± 9.9	90.0	12	13.5	−	+
Ankjærgaard [23]	Cohort	201	COPD exacerbation/FEV_1_ 35.0 (32.7–37.3)	71.5 (69.9–73.0)	43.3	24	43.8	−	−
Bélanger [24]	Cohort	479	COPD exacerbation, GOLD I-IV/FEV_1_ 51.2 ± 16.8	68.9 ± 9.4	52.0	12	10.4	−	−
Bloom [25]	Cohort	54,990	COPD, GOLD I-IV/FEV_1_ n/a	Training: 69.9 ± 10.7; test: 70.0 ± 10.6	50.0	12	21.0	−	+
Cheng [26]	Cohort	429	COPD exacerbation/FEV_1_ n/a	76.2 ± 9.3	66.4	12	13.8	−	−
Coleta [27]	Cohort	78	COPD admitted to LTOT/FEV_1_ 40.7 ± 16.1	66.0 ± 8.9	55.1	12	15.4	−	−
Duenk [28]	Cohort	155	COPD exacerbation, GOLD 0-IV/29% had FEV_1_ < 30% of predicted	67.5 ± 9.6	68.0	12	19.4	−	+
Duman [29]	Cohort	1,704	COPD exacerbation/FEV_1_ n/a	Group 1: 70.0 (61.0–80.0); group 2: 71.0 (63.0–78.0)	65.5	6	15.0	−	−
Edwards [30]	Cohort	133	COPD exacerbation/FEV_1_ n/a	72.7 ± 10.0	51.1	12	26.0	−	−
Eriksen [31]	Cohort	300	COPD exacerbation, GOLD II-IV/FEV_1_ 34.9 (group 1) and 37.6 (group 2)	72.1 ± n/a	38.3	12	25.5	+	−
Fan [32]	Cohort	3,282	COPD exacerbation/FEV_1_ 62.4	65.6 ± 10.9	96.3	12	5.1	−	−
García-Sanz [33]	Cohort	757	COPD exacerbation, GOLD I-IV/FEV_1_ n/a	74.8 ± 11.2	77.0	12	26.2	+	−
Gavazzi [34]	Cohort	267	COPD/(FEV_1_ < 30% or on LTOT > 8 h/day)	75.0 ± 9.0	70.8	6	37.0	+	−
Gudmundsson [35]	Cohort	416	COPD, GOLD I-IV/FEV_1_ 40.6 ± 19.2 (survivors) and 33.5 ± 14.4 (dead)	Survivors: 68.2 ± 10.9; dead: 72.1 ± 8.7	48.1	24	29.3	+	−
Guerrero [36]	Cohort	378	COPD exacerbation, GOLD A-D/FEV_1_ 44.2 ± 16.9	71.4 ± 10.0	84.0	12	21.0	+	−
Hallin [37]	Cohort	261	COPD exacerbation, GOLD ≥ 1/FEV_1_ 41 ± 19 (survivors) and 33 ± 14 (dead)	Survivors: 68.0 ± 12.0; dead: 72.0 ± 9.0	51.7	24	19.0	+	−
Ho [38]	Cohort	4,204	COPD/FEV_1_ n/a	75.0 ± 11.0	73.0	12	22.0	+	−
Hoong [39]	Cohort	286	COPD/FEV_1_ 64.4 ± 19.6 (nourished group) and 60.8 ± 20.5 (malnourished group)	66.6 ± 11.0	67.8	12	18.7	−	−
Horita [40]	Cohort	607	COPD/FEV_1_ 27 ± 7	67.0 ± 6.0	63.9	24	16.8	−	+
Hu [41]	Cohort	343	COPD exacerbation/FEV_1_ 51.6 ± 20.9 (survivors) and 52.0 ± 16.2 (dead)	Survivors: 75.8 ± 9.9; dead: 80.0 ± 8.1)	65.0	12	16.6	+	−
Man [42]	Cohort	4,803	COPD/FEV < 90% but ≥ 55%	53.0 ± 7.0	63.0	24	n/a	−	+
Marin [43]	Cohort	3,633	COPD, GOLD I-IV/FEV_1_ 53.8 ± 19.4	66.4 ± 9.7	93.3	12	6.3	−	+
Martinez [44]	RCT	1,218	COPD/FEV_1_ 26.75 ± 7.20	67.1 ± 6.1	61.2	24	n/a	−	+
Martinez-Rivera [45]	Cohort	117	COPD exacerbation, GOLD II-IV/FEV_1_ 37.71 ± 12.7 (survivors) and 36.13 ± 10.6 (dead)	72.0 ± 9.1	93.2	12	22.2	+	−
Morales [46]	Cohort	54,879	COPD/FEV_1_ 59.5 ± 20.4	74.1 ± 10.3	53.8	24	10.5	−	+
Navarro [47]	Cohort	80	COPD, GOLD B-D/FEV_1_ 40 ± 16	73.4 ± 8.9	90.0	21.4 (12.6–24.7)	21.0	+	−
Neo [48]	Cohort	124	COPD, GOLD III-IV/FEV_1_ 35.9 ± 9.8	71.7 ± 7.6	88.5	18	13.7	−	−
Niksarlioǧlu [49]	Cohort	49	COPD exacerbation, GOLD II-IV/FEV_1_ 34 ± 12	71.1 ± 10.9	77.6	24	14.9	+	−
Park [50]	Cohort	314	COPD exacerbation, with or without CAP/FEV_1_ 54.6–58.7 ± 21.0–23.9 with CAP), 55.5–60.7 ± 22.5–29.8 (without CAP)	72.2 ± 9.4	76.4	12	18.2	+	+
Pascual-Guardia [51]	Cohort	248	COPD exacerbation/FEV_1_ 35 (24–45) (group 1) and 40 (28–51) (group 2)	Group 1: 74.5 (67.0–80.0); group 2: 73.0 (63.0–80.0)	80.6	24	38.2	−	−
Philip [52]	Cohort	22,019	COPD/FEV_1_ n/a	73.0 ± 10.4	50.3	6	13.2	−	−
Pinto-Plata [53]	Cohort	198	COPD/FEV_1_ n/a	68.0 ± 9.0	85.0	24	42.0	+	−
Puhan [54]	Cohort	409	COPD, GOLD II-IV/FEV_1_ n/a	67.3 ± 10.0	57.0	24	9.3	−	+
Ranieri [55]	Cohort	244	COPD exacerbation or respiratory failure, GOLD I-III/FEV_1_ n/a	81.7 ± 7.3	44.7	6	20.0	+	−
Renom [56]	Cohort	116	COPD GOLD II-IV/FEV_1_ 36.5 ± 13.4	70.6 ± 8.6	94.0	24	36.0	+	−
Shin [57]	Cohort	134	COPD, GOLD A-D/FEV_1_ 55.0 + 20.4	72.8 ± 8.8	76.1	6	24.6	+	−
Slenter [58]	Cohort	260	COPD exacerbation, GOLD I-IV/FEV_1_ 45.0 ± 18.0	70.5 ± 10.8	50.0	12	27.7	+	−
Stolz [59]	Cohort	549	COPD, GOLD II-IV/FEV_1_ 48.9 ± 18.3	66.0 ± 11.4	69.8	24	7.8	+	−
Yohannes [60]	Cohort	100	COPD exacerbation/FEV_1_ 40 ± 15 (survivors) and 39 ± 14 (dead)	73.0 (60.0–98.0)	48.0	12	36.0	−	−
Zhan [61]	Cohort	418,251	COPD/FEV_1_ n/a	67.0 ± 13.0	60.0	12	5.8	−	+
Zimmermann [62]	Cohort	211	COPD, GOLD II-IV/FEV_1_ n/a	72.0 (64.0–77.0)	71.0	12	18.4	−	−

CAP, community-acquired pneumonia; COPD, chronic obstructive pulmonary disease; FEV_1_, forced expiratory volume in the first second; GOLD, Global Initiative for Chronic Obstructive Lung Disease; LTOT, long-term oxygen therapy; RCT, randomized controlled trial

**Table 2:** Summary of risk-of-bias assessment

Study	Study participation	Study attrition	Predictors	Outcome	Statistical analysis and cofounding	Performance of prediction tool	Overall bias
Abu Hussein, 2014	L	H	L	L	M	M	M
Almagro, 2014	L	H	L	L	M	M	M
Ankjærgaard, 2017	L	M	M	L	H	NA	M
Bélanger, 2018	L	H	M	L	L	NA	M
Bloom, 2019	L	H	L	L	L	L	L
Cheng, 2020	L	H	L	L	L	NA	L
Coleta, 2008	L	H	L	L	M	NA	M
Duenk, 2017	L	H	L	L	H	M	H
Duman, 2015	L	M	L	L	H	NA	M
Edwards, 2011	L	H	L	L	H	H	H
Eriksen, 2010	L	M	L	L	H	NA	M
Fan, 2002	M	H	M	L	L	M	H
García-Sanz, 2017	M	H	L	L	M	NA	M
Gavazzi, 2015	L	H	L	L	M	NA	M
Gudmundsson, 2006	L	M	L	L	H	NA	M
Guerrero, 2016	L	H	M	L	M	NA	M
Hallin, 2007	L	H	L	L	M	NA	M
Ho, 2014	L	H	L	L	M	NA	M
Hoong, 2017	M	H	M	M	M	NA	H
Horita, 2016	L	M	L	L	M	M	M
Hu, 2016	L	M	M	L	M	NA	M
Man, 2006	M	H	L	L	L	M	M
Marin, 2013	M	H	L	L	M	M	H
Martinez, 2008	M	M	L	L	L	M	M
Martinez-Rivera, 2012	L	H	M	L	M	NA	M
Morales, 2018	L	H	L	L	M	M	M
Navarro, 2015	L	H	L	L	M	H	H
Neo, 2017	L	H	L	L	M	M	M
Niksarlioǧlu, 2013	L	H	L	L	L	NA	L
Park, 2020	L	H	L	L	M	M	M
Pascual-Guardia, 2017	M	H	L	L	H	H	H
Philip, 2012	L	H	H	L	L	NA	M
Pinto-Plata, 2004	L	M	L	L	M	NA	L
Puhan, 2013	L	L	L	L	M	M	L
Ranieri, 2008	L	H	L	L	M	NA	M
Renom, 2010	L	H	L	L	M	NA	M
Shin, 2019	L	H	L	L	M	NA	M
Slenter, 2013	L	M	L	L	M	NA	L
Stolz, 2014	L	H	L	L	L	M	M
Yohannes, 2005	L	H	L	L	H	M	H
Zhan, 2020	L	H	L	L	H	L	M
Zimmermann, 2020	L	M	M	L	M	NA	M

H, high risk-of-bias; L, low risk-of-bias; M, moderate risk-of-bias; NA, not applicable

**Fig. 2:**
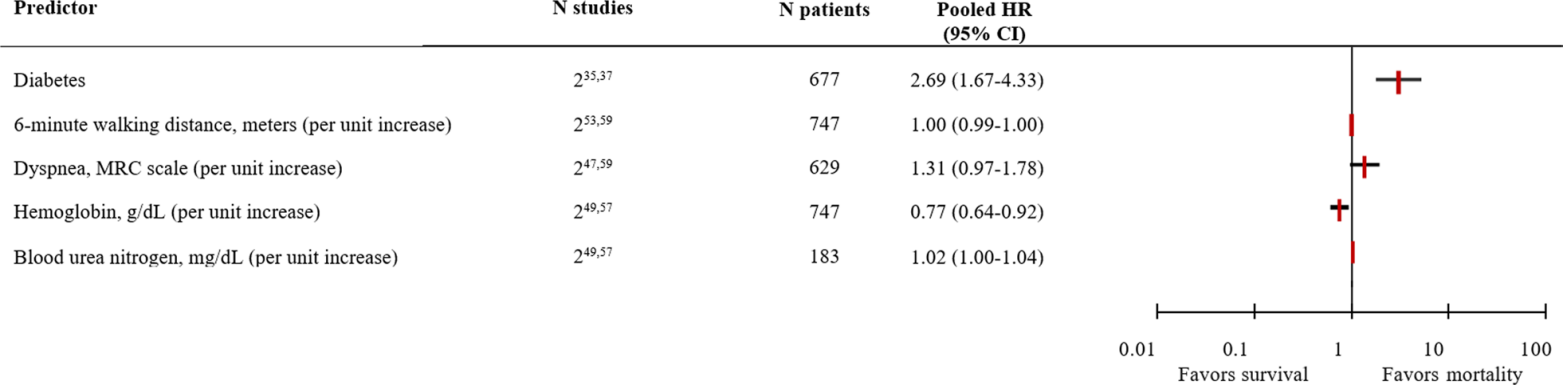
Summary forest plot of pooled hazard ratios for mortality with a fixed-effects model. CI, confidence interval; HR, hazard ratio; MRC, Medical Research Council

**Fig. 3:**
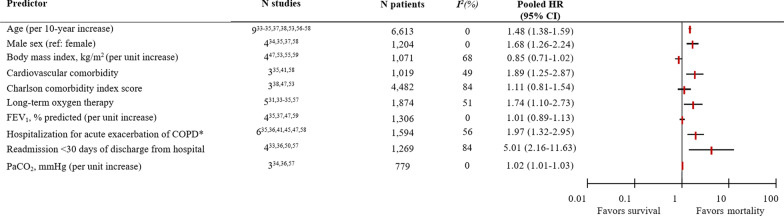
Summary forest plot of pooled hazard ratios for mortality with a random-effects model. CI, confidence interval; COPD, chronic obstructive pulmonary disease; FEV_1_, forced expiratory volume in one second; HR, hazard ratio; PaCO_2_, partial pressure of carbon dioxide in the arterial blood. *In the previous 12 or 24 months

Five potential predictors were studied with the fixed-effects model due to the low number of studies (Fig. 2). Of those predictors, having diabetes (HR 2.69; 95% CI 1.67–4.33) significantly increased the likelihood that a patient would die. Additionally, lower hemoglobin levels (HR 0.77; 95% CI 0.64–0.92) significantly decreased the likelihood of survival. Six-minute walking distance, dyspnea, and blood urea nitrogen were not significantly associated with mortality. Six of the 10 potential predictors that were analyzed with the random-effects model were significantly associated with mortality (Fig. 3 and Additional file 1: e-Fig. S1). Hospitalization for acute exacerbation of COPD in the previous 12 or 24 months (pooled from 6 studies; HR 1.97; 95% CI 1.32–2.95) and readmission within 30 days of discharge from the hospital (pooled from 4 studies; HR 5.01; 95% CI 2.16–11.63) significantly increased the risk of mortality. Other significant predictors were age, male sex, and long-term oxygen therapy. The presence of cardiovascular comorbidity was also significantly associated with mortality, but the Charlson comorbidity index score was not. Body mass index, FEV_1_, and partial pressure of carbon dioxide in the arterial blood (PaCO_2_) were also not significantly associated with mortality. Due to the limited number of low risk-of-bias studies, a meta-analysis with those studies only was not possible. The between-study heterogeneity was insignificant, i.e. *I*^2^ 0%, for predictors age, male sex, FEV_1_, or PaCO_2_. There was moderate heterogeneity, i.e. *I*^2^ 40–60%, for cardiovascular comorbidity, long-term oxygen therapy, or hospitalization for acute exacerbation of COPD. Predictors that showed substantial between-study heterogeneity, i.e. *I*^2^  > 60%, were body mass index, Charlson comorbidity index score, or readmission within 30 days of discharge. Funnel plots showed no evidence of major publication bias (Additional file 1: e-Fig. S2). A list of the variables that were excluded from the meta-analysis is presented in Additional file 1: e-Table S3.

Eleven studies reported on 19 different multicomponent prognostic models for mortality within a period of 3–24 months, of which the ADO, BODE, BODEX, CODEX, and DOSE models were most studied. (Table 3 and Additional file 1: e-Table S4). These models partly included overlapping variables (e.g. FEV_1_, body mass index, dyspnea, previous exacerbations) in various combinations. All prognostic models had a moderate discriminative ability with an AUC or c-statistic ranging between 0.6–0.8 (Table 3). The models were studied for various follow-up periods, but showed comparable discriminative abilities. Model calibration was not reported for the prognostic models.

**Table 3:** Summary of the performance of the multicomponent prognostic models

Prognostic tool	Variables	Study	N	Discriminative ability	3-month mortality	6-month mortality	12-month mortality	24-month mortality
ADO	Age, dyspnea (MRC), airflow obstruction (FEV_1_)	Almagro, 2014	606	AUC	0.651 (0.618–0.682)	–	0.641 (0.609–0.673)	–
		Marin, 2013	3633	c-statistic	–	0.701	0.702	–
		Bloom, 2019	54,990	AUC			T: 0.675 (0.655–0.694) V: 0.568 (0.514–0.595)	
		Morales, 2018	54,879	c-statistic	–	–	0.737 (0.727–0.746)	0.741 (0.735–0.748)
		Puhan, 2013	409	AUC	–	–	–	0.80 (0.71–0.89)
		Horita, 2016	607	AUC	–	–	–	0.67
ADO + handgrip strength		Puhan, 2013	409	AUC	–	–	–	0.80 (0.71–0.89)
ADO + sit-to-stand test		Puhan, 2013	409	AUC	–	–	–	0.82 (0.74–0.90)
BARC	Body mass index and blood results, Age, Respiratory Variables (Airflow obstruction, Exacerbations, Smoking), Comorbidities	Bloom, 2019	54,990	c-statistic			T: 0.794 (0.782–0.807) V: 0.671 (0.647–0.695)	
BOD	Body mass index, Airflow obstruction (FEV_1_), Dyspnea (mMRC)	Puhan, 2013	409	AUC	–	–	–	0.66 (0.55–0.77)
BODE	Body mass index, Airflow obstruction (FEV_1_), Dyspnea (mMRC), Exercise capacity (6MWD)	Marin, 2013	3633	c-statistic	–	0.680	0.682	–
		Horita, 2016	607	AUC	–	–	–	0.67
BODE + handgrip strength		Puhan, 2013	409	AUC	–	–	–	0.68 (0.57–0.80)
BODE + sit-to-stand test		Puhan, 2013	409	AUC	–	–	–	0.78 (0.70–0.87)
eBODE	BODE + previous exacerbations	Marin, 2013	3633	c-statistic	–	0.680	0.683	–
mBODE	Body mass index, Airflow obstruction (FEV_1_), Dyspnea (UCSD)	Martinez, 2008	1218	c-statistic	–	0.66	0.68	0.69
BODEX	Body mass index, Airflow obstruction (FEV_1_), Dyspnea (mMRC), Previous exacerbations	Almagro, 2014	606	AUC	0.615 (0.582–0.647)	–	0.615 (0.582–0.648)	–
		Marin, 2013	3633	c-statistic	–	0.651	0.651	–
		Bloom, 2019	54,990	AUC			T: 0.483 (0.453–0.512) V: 0.413 (0.379–0.447)	
CODEX	Comorbidity, Airflow obstruction (FEV_1_), Dyspnea (mMRC), Previous exacerbations	Almagro, 2014	606	AUC	0.724 (0.693–0.753)	–	0.679 (0.647–0.709)	–
		Morales, 2018	54,879	c-statistic	–	–	0.671 (0.661–0.682)	0.676 (0.668–0.683)
COPD Prognostic Score	Age, Airflow obstruction (FEV_1_), Dyspnea (mMRC), Hemoglobin, Activity (Daily Activity Scale), Emergency admissions (last 24 months)	Horita, 2016	607	AUC	–	–	–	0.72
DOSE	Dyspnea (mMRC), Airflow obstruction (FEV_1_), Smoking status, Previous exacerbations	Almagro, 2014	606	AUC	0.601 (0.568–0.633)	–	0.595 (0.562–0.628)	–
		Marin, 2013	3633	c-statistic	–	0.632	0.631	–
		Bloom, 2019	54,990	AUC			T: 0.591 (0.568–0.614) V: 0.515 (0.485–0.546)	
		Morales, 2018	54,879	c-statistic	–	–	0.669 (0.658–0.679)	0.672 (0.664–0.679)
ProPal-COPD	Body mass index, Airflow obstruction (FEV_1_), Dyspnea (mMRC), Comorbidity, Previous exacerbations, Surprise question, Clinical COPD questionnaire	Duenk, 2017	155	AUC	–	–	0.82 (0.81–0.82)	–
SAFE	Airflow obstruction (FEV_1_), Exercise capacity (6MWD), Quality of life (SGRQ)	Marin, 2013	3633	c-statistic	–	0.641	0.642	–
Unnamed model 1	Age, Body mass index, Race, C-reactive protein	Man, 2006	4803	c-statistic	–	–	0.82	–
Unnamed model 2	Readmission ≤ 30 days, Community-acquired pneumonia	Park, 2020		AUC		0.678 (0.597–0.758)		
Unnamed model 3	Age, Sex	Zhan, 2020		c-statistic			T: 0.771 (0.767–0.775) V: 0.768 (0.764–0.772)	

6MWD, 6-min walking distance; FEV_1_, forced expiratory volume in one second; mMRC, Modified Medical Research Council; PaO_2_/FiO_2_, ratio of arterial oxygen partial pressure to fractional inspired oxygen; SGRQ, St. George's Respiratory Questionnaire; Surprise question, ‘Would you be surprised if this patient died in the next year?’; T, test set; UCSD, University of California San Diego–Shortness of Breath Questionnaire; V, external validation set

## Discussion

This systematic review and meta-analysis aimed to identify predictors of mortality within 3–24 months in patients with COPD. We found eight predictors that were significantly associated with mortality. Four of these predictors are related to patient demographics or history (age, sex, diabetes, and cardiovascular comorbidity), three to the underlying pulmonary disease (long-term oxygen therapy, previous hospitalization for acute exacerbation of COPD, and readmission within 30 days), and one to laboratory tests (haemoglobin). Overall, all significant predictors in our study seem to be readily obtainable, by taking the patient’s medical history and by performing simple blood tests. Similar to our findings, a review by Singanayagam et al. [7] found that age, diabetes, and cardiovascular comorbidity were associated with mortality within 3–24 month [7]. However, they found sex and long-term oxygen therapy to be associated with 3-month mortality and not with 3–24 month mortality as it is the case in our study. Further, low body mass was associated with mortality during longer follow-ups of 4-year and 17-year in studies by, respectively, Landbo et al. [15] and Schols et al. [16]. Additionally, dyspnea severity, which was not found to be a significant predictor in our study, was associated with 5-year mortality in a study by Nishimura et al. [17].

We also reviewed existing prognostic models for prediction of mortality in patients with COPD. The studies that reported on prognostic models did not provide the relevant effect sizes and associated standard errors of the individual variables. Therefore, the variables from those studies could not be included in our meta-analysis. Interestingly, some of the predictors included in prognostic models were not significant in our meta-analysis, such as dyspnea, body mass index, Charlson comorbidity index, 6-min walking distance, and FEV_1_. Of the predictors that were significantly associated with mortality in our meta-analysis, only age, previous hospitalization for acute exacerbation of COPD, and hemoglobin level were also included in one or more prognostic models, namely the ADO, BODEX, CODEX, COPD Prognostic Score, or ProPal-COPD models. Overall, the majority of the existing prognostic models had a moderate discriminative ability (AUC 0.6–0.8). Additionally, in a meta-analysis of the 6-min walking distance in 14,497 patients with COPD, Celli et al. (2016) found an AUC to predict mortality of 0.71 and 0.70 at 6 and 12 months, respectively [18]. No study reported on the calibration of the models, which is needed to judge their applicability in a specific clinical setting. Studies validating prognostic models for predicting mortality in COPD and their methodological quality should therefore be improved.

The surprise question (‘Would you be surprised if this patient died in the next year?’), which is usually recommended to be used to identify patients who are likely to die within a period of 12 months, has not been thoroughly studied for COPD yet [19]. The surprise question was only included in the ProPal-COPD model [20]. This model was one of the two models with a good discriminative ability (AUC > 0.8). Although this finding should be interpreted with some caution due to the small sample size of the study, it suggests that the physician’s clinical judgement might be important in the prediction of mortality.

This systematic review had several limitations. Firstly, some variables were pooled from only two studies, which is not ideal for a meta-analysis. We could not pool some studies in the meta-analysis because of incomplete reporting of data. Additionally, some variables could not be included in the meta-analysis because the studies did not use uniform methods for categorization of the outcomes. Furthermore, due to the low number of studies that had a low risk-of-bias based on our customized appraisal tool, we could not perform a meta-analysis with only low risk-of-bias studies. In addition, the quality of the studies was limited by the lack of data about the loss to follow-up and handling of missing values. Studies on prognostic factors could decrease study bias by reporting the number of missing values, how those values were analyzed, and the number of patients lost to follow-up. Secondly, there was substantial heterogeneity across studies for the predictors body mass index, Charlson comorbidity index, and readmission, which may be caused by the different follow-up periods, ranging between 6 and 24 months, and different study populations regarding measured FEV_1_ levels. Additionally, for the Charlson comorbidity index, the heterogeneity could be especially explained by the results from the small study of Navarro et al., which were discrepant to the results of the larger ones, possibly indicating selection bias. Although the *I*^2^, which is an indicator for statistical heterogeneity, was insignificant or moderate for most predictors, the pooled overall prediction effect should be interpreted with caution. Lastly, we only included published studies, especially studies from 2000 onward, whereby we might have missed some predictors of mortality.

## Conclusion

This systematic review and meta-analysis provide an overview of predictors and multicomponent prognostic models for mortality within 3–24 months for patients with COPD. We conclude that mortality within 3–24 months is to a certain extent predictable. The existing models showed overall moderate discriminative ability, but no information on model calibration was available. We therefore suggest that there is a need for improvement in the validation of prognostic models. A more accurate prediction of mortality might give physicians more certainty in timely initiating ACP in patients with COPD. Further prognostic research should include physician’s clinical prediction of mortality based on the ‘surprise question’.

## Supplementary Information

**Additional file 1**. Supplementary tables and figures.

## Data Availability

The data is included in the Supplementary Material and will not be shared separately.
