# Supplementary material for: Predictors of mortality in chronic obstructive pulmonary disease: a systematic review and meta-analysis
Source: BMC Pulm Med. 2022 Apr 4;22:125. doi: 10.1186/s12890-022-01911-5 (PMC8978392; doi:10.1186/s12890-022-01911-5)
Supplement: Supplementary file 1 — Additional file 1. Supplementary tables and figures. [file 12890_2022_1911_MOESM1_ESM.docx]

# Additional file 1: supplementary tables and figures

## e-Table S1 Search strategies developed for the online databases

| ***Embase***  **(**'chronic disease'/de OR 'lung fibrosis'/exp OR 'obstructive airway disease'/exp OR 'neoplasm'/exp OR (((chronic* OR advance* OR palliative) NEAR/4 (ill* OR diseas* OR condition* OR lung OR pulmonar* OR cancer* OR neoplas* OR tumor* OR tumour* OR patient*)) OR ((lung OR pulmonar* OR airway) NEAR/3 (fibros* OR obstruct*)) OR COPD OR asthma* OR bronchitis):ab,ti**) AND** ('physical disease by body function'/de OR 'health status indicator'/exp OR 'cognition'/de OR 'hospital admission'/de OR 'hospital readmission'/de OR (condition OR conditions OR cognit* OR admission* OR readmission* OR ((symptom*) NEAR/3 (sign* OR digest* OR respirat* OR pathol*))):ab,ti) **AND** ('terminal care'/exp OR 'death'/exp OR ('end of life' OR ((mors* OR mortalit* OR died OR die OR dying OR death OR fatal*) NEAR/6 (year OR month OR months OR week OR weeks OR days OR time*)) OR ((end *OR EOL* OR palliat* OR terminal* OR final OR last) NEAR/3 (care OR phase* OR stage* OR month* OR week* OR year))):ab,ti) **AND** ('prediction'/de OR 'predictive value'/de OR 'prognosis'/exp OR 'prognostic assessment'/exp OR ('surprise question' OR ((predict* OR indicat* OR prognost*) NEAR/4 (value* OR factor* OR tool* OR index OR indices OR assess*)) OR ((early OR time*) NEXT/1 (identificat*))):ab,ti) **AND** english:la **AND (**'article'/it OR 'article in press'/it OR 'note'/it OR 'review'/it**)** |
| --- |
| ***PubMed***  **(**((chronic*[tiab] OR advance*[tiab] OR palliative[tiab]) AND (ill[tiab] OR illness[tiab] OR diseas*[tiab] OR condition*[tiab] OR lung[tiab] OR pulmonar*[tiab] OR cancer*[tiab] OR neoplas*[tiab] OR tumor*[tiab] OR tumour*[tiab] OR patient[tiab] OR patients[tiab])) OR ((lung[tiab] OR pulmonar*[tiab] OR airway[tiab]) AND (fibros*[tiab] OR obstruct*[tiab])) OR COPD[tiab] OR asthma*[tiab] OR bronchitis[tiab]**) AND (**condition[tiab] OR conditions[tiab] OR cognit*[tiab] OR admission*[tiab] OR readmission*[tiab] OR ((symptom*[tiab]) AND (sign[tiab] OR signs[tiab] OR digest*[tiab] OR respirat*[tiab] OR pathol*[tiab]))**)** **AND (**end of life[tiab] OR ((mors*[tiab] OR mortalit*[tiab] OR died[tiab] OR die[tiab] OR dying[tiab] OR death[tiab] OR fatal*[tiab]) AND (year[tiab] OR month[tiab] OR months[tiab] OR week[tiab] OR weeks[tiab] OR days[tiab] OR time*[tiab])) OR ((end[tiab] *OR EOL*[tiab] OR palliat*[tiab] OR terminal*[tiab] OR final[tiab] OR last[tiab]) AND (care[tiab] OR phase*[tiab] OR stage*[tiab] OR month*[tiab] OR week*[tiab] OR year[tiab]))) **AND (**surprise question[tiab] OR ((predict*[tiab] OR indicat*[tiab] OR prognost*[tiab]) AND (value*[tiab] OR factor*[tiab] OR tool*[tiab] OR index[tiab] OR indices[tiab] OR assess*[tiab])) OR early identif*[tiab] OR timely identif*[tiab]**)** **AND** publisher[sb] |
| ***MEDLINE***  **(**"Chronic Disease"/ OR exp "Pulmonary Fibrosis"/ OR exp "Lung Diseases, Obstructive"/ OR exp "Neoplasms"/ OR (((chronic* OR advance* OR palliative) ADJ4 (ill* OR diseas* OR condition* OR lung OR pulmonar* OR cancer* OR neoplas* OR tumor* OR tumour* OR patient*)) OR ((lung OR pulmonar* OR airway) ADJ3 (fibros* OR obstruct*)) OR COPD OR asthma* OR bronchitis).ab,ti.**) AND** ("Signs and Symptoms"/ OR exp "Health Status Indicators"/ OR "Cognition"/ OR exp "Hospitalization"/ OR (condition OR conditions OR cognit* OR admission* OR readmission* OR ((symptom*) ADJ3 (sign* OR digest* OR respirat* OR pathol*))).ab,ti.) **AND** (exp "Terminal Care"/ OR exp "Death"/ OR ("end of life" OR ((mors* OR mortalit* OR died OR die OR dying OR death OR fatal*) ADJ6 (year OR month OR months OR week OR weeks OR days OR time*)) OR ((end *OR EOL* OR palliat* OR terminal* OR final OR last) ADJ3 (care OR phase* OR stage* OR month* OR week* OR year))).ab,ti.) **AND** ("Predictive Value of Tests"/ OR "Prognosis"/ OR ("surprise question" OR ((predict* OR indicat* OR prognost*) ADJ4 (value* OR factor* OR tool* OR index OR indices OR assess*)) OR ((early OR time*) ADJ1 (identificat*))).ab,ti.) **AND** English.lg. **AND** Journal Article.pt. |
| ***Web of Science***  **TS=((**(((chronic* OR advance* OR palliative) NEAR/3 (ill* OR diseas* OR condition* OR lung OR pulmonar* OR cancer* OR neoplas* OR tumor* OR tumour* OR patient*)) OR ((lung OR pulmonar* OR airway) NEAR/2 (fibros* OR obstruct*)) OR COPD OR asthma* OR bronchitis)**) AND** ((condition OR conditions OR cognit* OR admission* OR readmission* OR ((symptom*) NEAR/2 (sign* OR digest* OR respirat* OR pathol*)))) **AND** (("end of life" OR ((mors* OR mortalit* OR died OR die OR dying OR death OR fatal*) NEAR/6 (year OR month OR months OR week OR weeks OR days OR time*)) OR ((end *OR EOL* OR palliat* OR terminal* OR final OR last) NEAR/2 (care OR phase* OR stage* OR month* OR week* OR year)))) **AND** (("surprise question" OR ((predict* OR indicat* OR prognost*) NEAR/2 (value* OR factor* OR tool* OR index OR indices OR assess*)) OR ((early OR time*) NEAR/1 (identificat*))))**) AND LA=** English **AND** **DT=**Article |
| ***Cochrane Central***  **(**(((chronic* OR advance* OR palliative) NEAR/4 (ill* OR diseas* OR condition* OR lung OR pulmonar* OR cancer* OR neoplas* OR tumor* OR tumour* OR patient*)) OR ((lung OR pulmonar* OR airway) NEAR/3 (fibros* OR obstruct*)) OR COPD OR asthma* OR bronchitis):ab,ti**) AND** ((condition OR conditions OR cognit* OR admission* OR readmission* OR ((symptom*) NEAR/3 (sign* OR digest* OR respirat* OR pathol*))):ab,ti) **AND** (('end of life' OR ((mors* OR mortalit* OR died OR die OR dying OR death OR fatal*) NEAR/6 (year OR month OR months OR week OR weeks OR days OR time*)) OR ((end *OR EOL* OR palliat* OR terminal* OR final OR last) NEAR/3 (care OR phase* OR stage* OR month* OR week* OR year))):ab,ti) **AND** (('surprise question' OR ((predict* OR indicat* OR prognost*) NEAR/4 (value* OR factor* OR tool* OR index OR indices OR assess*)) OR ((early OR time*) NEXT/1 (identificat*))):ab,ti) |

## e-Table S2 Domains and items for risk-of-bias assessment

| Domain | Items | Total domain points |
| --- | --- | --- |
|  |  |  |
| Study participation | 1. Recruitment procedure 2. Study population 3. In- and exclusion criteria 4. Baseline study characteristics | 8 |
| Study attrition | 1. Number of loss to follow-up 2. Reasons for loss to follow-up 3. Differences between completers and non-completers 4. Handling of missing data | 8 |
| Predictors | 1. Definition of predictor(s) 2. Measurement of predictor(s) 3. Handling of predictor(s) 4. Data presentation | 8 |
| Outcome | 1. Outcome definition 2. Outcome presentation | 4 |
| Statistical analysis and confounding | 1. Method of selection of variables 2. Occurrence of mortality in relation to predictors 3. Presentation of crude variables (univariate analysis) 4. Presentation of adjusted variables (multivariate analysis) 5. Account for potential confounders 6. Selective reporting | 12 |
| Performance of prediction tool | 1. Tool performance 2. Internal validation 3. External validation | 6 |

Scoring per item: 2 points: bias not present; 1 point: bias possibly present; 0 points: bias present.

Scoring per domain: low risk-of-bias: ≥80% of the total domain points; moderate risk-of-bias: 60-79% of the total domain points; high risk-of-bias: <60% of the total domain points.

## e-Figure S1 Forest plots per individual predictor using random-effects model

 **Age (per 10-year increase)**

0.01 0.1 1 10 100

Favors survival Favors mortality

| **Study** | **N** |  | **HR  (95% CI)** |
| --- | --- | --- | --- |
| Ho, 2014 | 4,204 |  | 1.48 (1.34-.163) |
| Slenter, 2013 | 260 |  | 1.48 (1.11-1.98) |
| Renom, 2010 | 116 |  | 1.22 (0.82-1.80) |
| Gavazzi, 2015  a | 267 |  | 2.37 (1.31-4.28) |
| Hallin, 2007 | 261 |  | 1.30 (0.90-1.87) |
| Garcia-Sanz, 2017 | 757 |  | 1.63 (1.35-1.97) |
| Shin, 2019 | 134 |  | 1.52 (0.90-2.57) |
| Gudmundsson, 2006 | 416 |  | 1.49 (1.17-1.90) |
| Pinto-Plata, 2004 | 198 |  | 1.34 (1.06-1.71) |
| **Total (95% CI)**  *I*^2^ = 0 |  |  | **1.48 (1.38-1.59)** |

**Male sex (ref: female)**

| **Study** | **N** |  | **HR  (95% CI)** |
| --- | --- | --- | --- |
| Slenter, 2013 | 260 |  | 2.00 (1.15-3.48) |
| Gavazzi, 2015 | 267 |  | 1.83 (0.73-4.60) |
| Hallin, 2007 | 261 |  | 1.67 (0.85-3.25) |
| Gudmundsson, 2006 | 416 |  | 1.49 (0.98-2.28) |
|  |  |  |  |
| **Total (95% CI)**  *I*^2^ = 0 |  |  | **1.68 (1.26-2.24)** |

0.01 0.1 1 10 100

Favors survival Favors mortality

**Body mass index, kg/m^2^ (per unit increase)**

| **Study** | **N** |  | **HR  (95% CI)** |
| --- | --- | --- | --- |
| Ranieri, 2008 | 244 |  | 0.85 (0.73-0.99) |
| Navarro, 2015 | 80 |  | 1.78 (0.40-7.90) |
| Stolz, 2014 | 549 |  | 0.56 (0.38-0.83) |
| Pinto-Plata, 2004 | 198 |  | 0.95 (0.91-0.99) |
|  |  |  |  |
| **Total (95% CI)**  *I*^2^ = 68 |  |  | **0.85 (0.71-1.02)** |

0.01 0.1 1 10 100

Favors survival Favors mortality

**Cardiovascular comorbidity**

| **Study** | **N** |  | **HR  (95% CI)** |
| --- | --- | --- | --- |
| Slenter, 2013 | 260 |  | 1.75 (1.03-2.97) |
| Hu, 2016 | 343 |  | 2.99 (1.67-5.36) |
| Gudmundsson, 2006 | 416 |  | 1.43 (0.92-2.23) |
|  |  |  |  |
| **Total (95% CI)**  *I*^2^ = 49 |  |  | **1.89 (1.25-2.87)** |

0.01 0.1 1 10 100

Favors survival Favors mortality

*Continuation of e-Figure S1*

 **Charlson comorbidity index score**

0.01 0.1 1 10 100

Favors survival Favors mortality

| **Study** | **N** |  | **HR  (95% CI)** |
| --- | --- | --- | --- |
| Ho, 2014 | 4,204 |  | 1.06 (1.03-1.09) |
| Navarro, 2015 | 80 |  | 3.25 (1.49-7.07) |
| Pinto-Plata, 2004 | 198 |  | 0.84 (0.68-1.03) |
|  |  |  |  |
| **Total (95% CI)**  *I*^2^ = 84 |  |  | **1.11 (0.81-1.54)** |

 **Long-term oxygen therapy**

0.01 0.1 1 10 100

Favors survival Favors mortality

| **Study** | **N** |  | **HR  (95% CI)** |
| --- | --- | --- | --- |
| Eriksen, 2010 | 300 |  | 3.00 (1.67-5.40) |
| Gavazzi, 2015 | 267 |  | 1.42 (0.61-3.29) |
| Garcia-Sanz, 2017 | 757 |  | 2.68 (1.16-6.17) |
| Shin, 2019 | 134 |  | 1.30 (0.56-3.03) |
| Gudmundsson, 2006 | 416 |  | 1.07 (0.62-1.84) |
|  |  |  |  |
| **Total (95% CI)**  *I*^2^ = 51 |  |  | **1.74 (1.10-2.73)** |

 **FEV_1_, % predicted (per unit increase)**

0.01 0.1 1 10 100

Favors survival Favors mortality

| **Study** | **N** |  | **HR  (95% CI)** |
| --- | --- | --- | --- |
| Navarro, 2015 | 80 |  | 1.34 (0.78-2.31) |
| Hallin, 2007 | 261 |  | 0.98 (0.77-1.25) |
| Stolz, 2014 | 549 |  | 1.15 (0.68-1.95) |
| Gudmundsson, 2006 | 416 |  | 0.98 (0.84-1.14) |
|  |  |  |  |
| **Total (95% CI)**  *I*^2^ = 0 |  |  | **1.01 (0.89-1.13)** |

**Hospitalization for acute exacerbation of COPD in the previous 12 or 24 months**

| **Study** | **N** |  | **HR  (95% CI)** |
| --- | --- | --- | --- |
| Martinez-Rivera, 2012 | 117 |  | 5.87 (1.30-26.52) |
| Slenter, 2013 | 260 |  | 2.56 (1.52-4.31) |
| Navarro, 2015 | 80 |  | 1.64 (0.78-3.44) |
| Guerrero, 2016 | 378 |  | 3.62 (1.70-7.70) |
| Hu, 2016 | 343 |  | 1.38 (0.79-2.41) |
| Gudmundsson, 2006 | 416 |  | 1.22 (0.79-1.89) |
|  |  |  |  |
| **Total (95% CI)**  *I*^2^ = 56 |  |  | **1.97 (1.32-2.95)** |

0.01 0.1 1 10 100

Favors survival Favors mortality

*Continuation of e-Figure S1*

**Readmission <30 days of discharge from the hospital**

| **Study** | **N patients** | **FU (mo.)** | **HR  (95% CI)** |
| --- | --- | --- | --- |
| Guerrero, 2016 | 378 | 12 | 2.48 (1.10-5.59) |
| Garcia-Sanz, 2017 | 757 | 12 | 2.32 (1.30-4.16) |
| Shin, 2019 | 134 | 6 | 14.78 (6.26-34.88) |
| Park, 2020 | 314 | 6 | 7.88 (4.55-13.63) |
|  |  |  |  |
| **Total (95% CI)**  *I^2^ = 84* |  |  | **5.01 (2.16-11.63)**  0.01 0.1 1 10 100  Favors survival Favors mortality |

 **PaCO_2_, mmHg (per unit increase)**

| **Study** | **N** |  | **HR  (95% CI)** |
| --- | --- | --- | --- |
| Gavazzi, 2015 | 267 |  | 1.03 (1.00-1.06) |
| Guerrero, 2016 | 378 |  | 1.02 (1.01-1.04) |
| Shin, 2019 | 134 |  | 1.02 (0.99-1.04) |
|  |  |  |  |
| **Total (95% CI)**  *I*^2^ = 0 |  |  | **1.02 (1.01-1.03)** |

0.01 0.1 1 10 100

Favors survival Favors mortality

e-Table S3 Variables excluded from the meta-analysis

| **Variable type** | **Variable** | **Number of studies** |
| --- | --- | --- |
| Age | - Age ≥90 vs ≤59   Age 80-89 vs ≤59  Age 70-79 vs ≤59  Age 60-69 vs ≤59  Age ≥67 vs <67 | 2^1,2^ |
| Body mass index | - Body mass index <20 kg/m2 vs 25-30   Body mass index 20-25 kg/m2 vs 25-30  Body mass index >30 kg/m2 vs 25-30 | 1^3^ |
| Co-medication | - Beta-blockers   Beta blocker at discharge  Nebulized beta-2-agonists and/or ipratropium  Both long-acting beta-agonist and inhaled corticosteroid  Long-acting beta-agonist without inhaled corticosteroid  inhaled corticosteroid without long-acting beta-agonist  Short acting beta-2-agonists metered-dose inhaler  Treatment with anti-dysrhythmic drugs | 4^4-7^ |
|  | - Statin at discharge | 1^7^ |
|  | - Ipratropium | 1^6^ |
|  | - Theophylline | 1^6^ |
| Comorbidity | - Atrial fibrillation | 1^8^ |
|  | - Hyperlipidemia | 1^7^ |
|  | - Liver cirrhosis | 1^7^ |
|  | - Dementia | 1^8^ |
|  | - Malignancy | 1^7^ |
| GOLD classification | - GOLD stage III VS stage I-II   GOLD stage IV VS stage I-II  GOLD stage I, II, and III VS stage IV  GOLD group C-D | 3^6,9,10^ |
| Laboratory variables | - Renal dysfunction: glomerular filtration rate | 1^2^ |
|  | - Anemia: hemoglobin <12 mg/dL (women) or <13 mg/dL (men) | 1^11^ |
|  | - Pro-adrenomedullin | 1^12^ |
|  | - C-reactive protein mg/L | 1^2^ |
|  | - High sensitive C-reactive protein, mg/dL | 1^9^ |
|  | - HSP27 (≥ 3098 pg/ml) | 1^10^ |
|  | - D-dimer >985 ug/L | 1^2^ |
|  | - Urea, mmol/l ≥8.0 | 1^13^ |
|  | - Sodium (for each 1 mEq/l increase) | 1^5^ |
|  | - Eosinophilia ≥200 cells/uL or ≥2% | 1^14^ |
|  | - Albumin (g/dL) | 1^15^ |
|  | - B-type natriuretic peptide (pg/mL) | 1^15^ |
|  | - NT-proBNP (age-adjusted) | 1^10^ |
|  | - Delta neutrophil index, % | 1^9^ |
| Exercise capacity | - 1-min Sit-to-stand test repetitions 19,5 +/- 8,7 vs 11,8 +/- 6,3 per 1 more repetition   1-min Sit-to-stand test repetitions 19,5 +/- 8,7 vs 11,8 +/- 6,3 per 5 more repetitions | 1^16^ |
|  | - Handgrip strength (kilogram-force)   Handgrip strength 36,4 +/-12,2 kg vs 30,1 +/-9,9 kg per 1 more kg  Handgrip strength 36,4 +/-12,2 kg vs 30,1 +/-9,9 kg per 5 more kg | 2^16,17^ |
|  | - 2MWD ≤80 m | 1^18^ |
| Pulmonary function | - FVC (% predicted) | 1^17^ |
| Pulmonary function | - FEV_1_ (% predicted) ≥27   FEV_1_ (% predicted) <27  FEV_1_ (L) | 2^1,19^ |
| Hospitalization-related | - Length of hospital stay per day   Mean stay (length of stay) | 2^7,8^ |
|  | - Being seen in the outpatient clinic | 1^4^ |
|  | - Intensive care unit admission   Intensive care unit treatment | 2^7,15^ |
| Dyspnea | - Baseline dyspnea index | 1^17^ |
|  | - mMRC dyspnea score 3, 4   mMRC dyspnea score ≥2 at admission  mMRC dyspnea score 0, 1, 2 | 2^1,20^ |
| Blood gas test | - PaCO_2_ >50 mmHg   PaCO_2_ ≥ 6.0 kPa | 2^2,13^ |
|  | - pH 7.20-7.35 vs ≥7.35   pH ≤7.20 vs ≥7.35 | 1^2^ |
|  | - PaO_2_ 60 mmHg vs ≥60   PaO_2_ (kPa) | 2^2,17^ |
| Scores / tools | - APACHE II score | 1^21^ |
|  | - Barthel index at discharge | 1^21^ |
|  | - BOD   BOD-A | 1^12^ |
|  | - BODE index (per unit increase)   BODE quartile 4 VS 1+2  BODE quartile 3 VS 1+2  mBODE: >1 point decrease  mBODE: >1 point increase  BODE-A | 4^5,12,22,23^ |
|  | - BODEX | 1^24^ |
|  | - CODEX | 1^24^ |
|  | - COPD Prognostic Score < 9   COPD Prognostic Score ≥ 9 | 1^1^ |
|  | - SGRQ Total per units - SGRQ Total score (%) - SGRQ score (4 units) Total score - SGRQ score (4 units) Activity - SGRQ score (4 units) Impact - SGRQ score (4 units) Symptoms | 3^3,6,17^ |
| Other | - Sputum culture: non-usual pathogen vs no pathogen   Sputum culture: usual pathogen vs no pathogen  Sputum culture: usual + non-usual pathogen vs no pathogen | 1^23^ |
|  | - Mechanical ventilation support | 1^25^ |
|  | - Current smoking | 1^6^ |

2MWD: 2-minutes walking distance; 6MWD: 6-minutes walking distance; APACHE: Acute Physiology and Chronic Health Evaluation; BOD: body mass index, airflow obstruction, dyspnea; BOD-A: body mass index, airflow obstruction (FEV_1_), dyspnea (mMRC), pro-adrenomedullin; BODE: body mass index, airflow obstruction (FEV_1_), dyspnea (mMRC), exercise capacity index (6MWD); BODE-A: body mass index, obstruction (FEV_1_), dyspnea (mMRC), exercise capacity (6MWD), pro-adrenomedullin; COPD: chronic obstructive pulmonary disease; FEV_1_: forced expiratory volume in one second; GOLD: Global Initiative for Chronic Obstructive Lung Disease; mBODE: body mass index, obstruction (FEV_1_), dyspnea (mMRC), exercise capacity (University of California San Diego–Shortness of Breath Questionnaire); mMRC: modified Medical Research Council; PaCO_2_: partial pressure of carbon dioxide in the arterial blood; PaO_2_: partial pressure of oxygen in the arterial blood; pH: potential hydrogen; SGRQ: St George's Respiratory Questionnaire

e-Figure S2 Funnel plots

Age (per 10-years increase) Male sex (ref: female) Body mass index, kg/m^2^ (per unit increase)


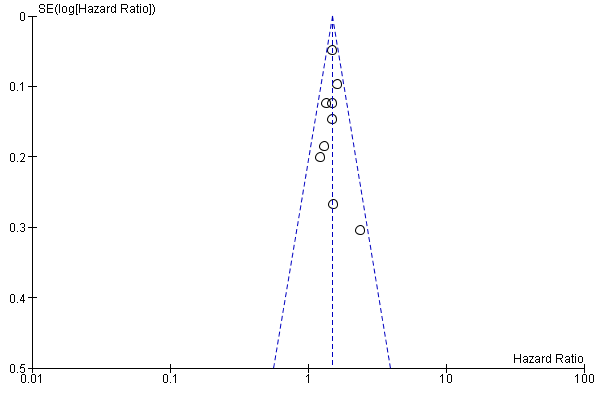

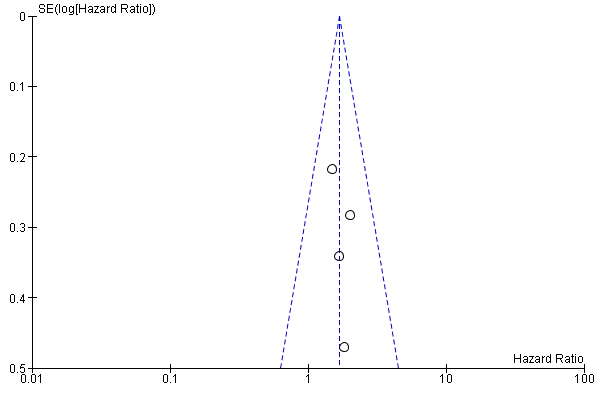

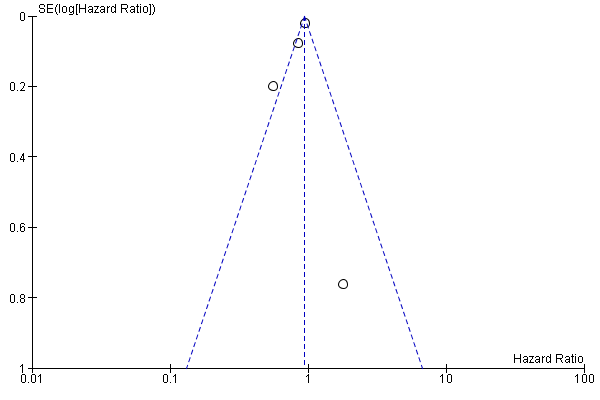


Cardiovascular comorbidity Charlson comorbidity index score Long-term oxygen therapy


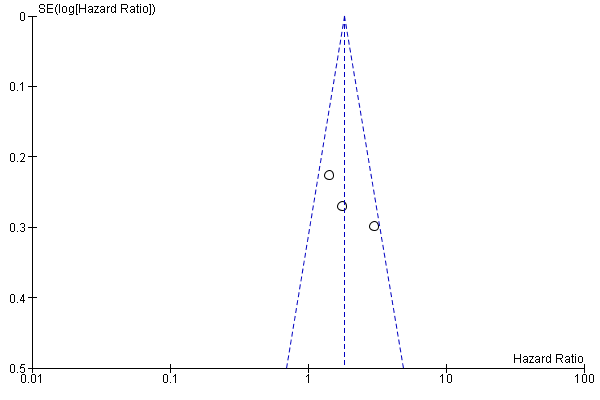

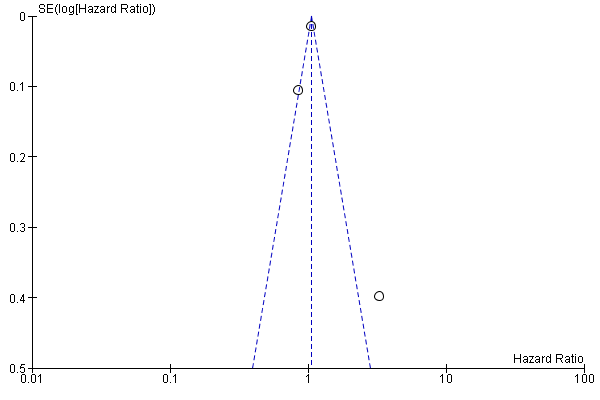

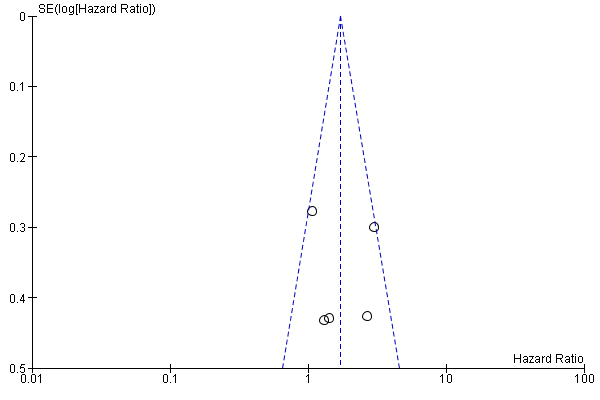


FEV1, % predicted (per unit increase) Hospitalization for AECOPD in previous 1 or 2 years Readmission <30 days


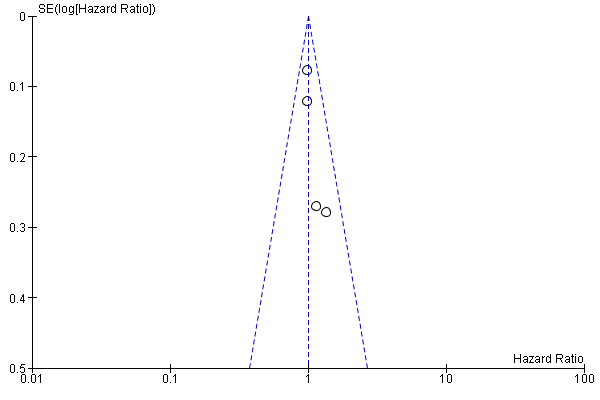

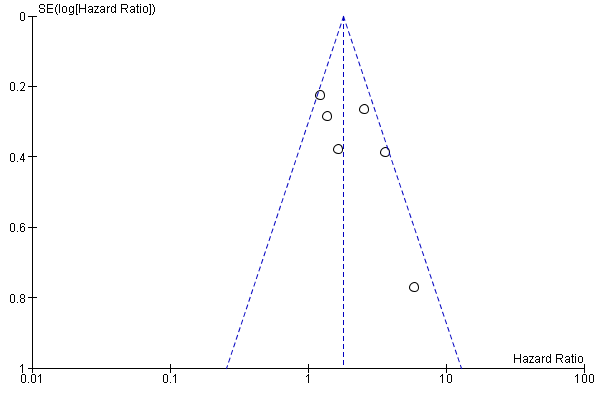

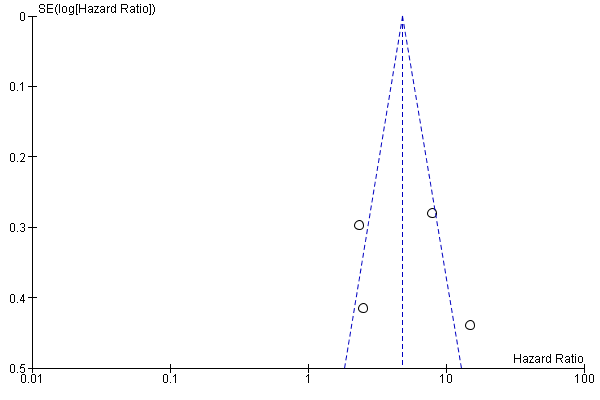


PaCO_2_, mmHg (per unit increase)


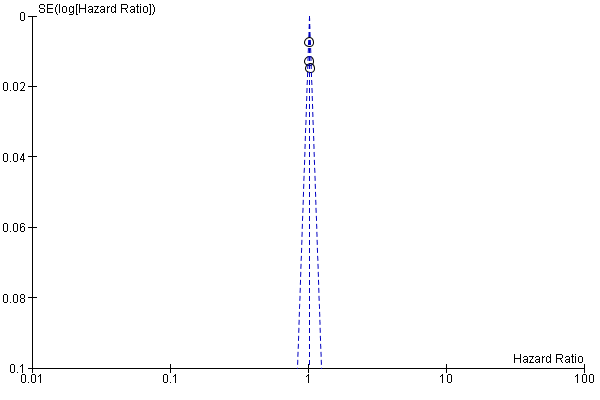


|  |  |  |
| --- | --- | --- |
|  |  |  |

e-Table S4 Summary of the variables of the prognostic models

| Prognostic model | Age | Airflow obstruction (FEV_1_) | Body mass index | Comorbidity | Dyspnea (mMRC scale) | Exercise capacity (6MWD) | Previous exacerbations | Other |
| --- | --- | --- | --- | --- | --- | --- | --- | --- |
| ADO | * | * |  |  | * |  |  | - |
| BARC | * | * | * | * |  |  | * | Smoking  Blood results |
| BOD |  | * | * |  | * |  |  | - |
| BODE |  | * | * |  | * | * |  | - |
| eBODE |  | * | * |  | * | * | * | - |
| *mBODE* |  | * | * |  | * | * |  | - |
| BODEX |  | * | * |  | * |  | * | - |
| CODEX |  | * |  | * | * |  | * | - |
| COPD Prognostic Score | * | * |  |  | * |  |  | Haemoglobin  Activity (Daily Activity Scale)  Emergency admissions (last 24 months) |
| DOSE |  | * |  |  | * |  | * | Smoking status |
| ProPal-COPD |  | * | * | * | * |  | * | Surprise question (‘Would you be surprised if this patient died in the next year?’)  Clinical COPD questionnaire |
| SAFE |  | * |  |  |  | * |  | Quality of life (SGRQ) |
| Unnamed model 1 | * |  | * |  |  |  |  | Race  C-reactive protein |
| Unnamed model 2 |  |  |  |  |  |  |  | Readmission ≤30 days  Community-acquired pneumonia |
| Unnamed model 3 | * |  |  |  |  |  |  | Sex |

Dyspnea in the mBODE was measured according to the University of California San Diego–Shortness of Breath Questionnaire.
6MWD: 6-minute walking distance; FEV_1_: forced expiratory volume in one second; mMRC: Modified Medical Research Council; SGRQ: St. George's Respiratory Questionnaire.

**References**

1. Horita N, Koblizek V, Plutinsky M, Novotna B, Hejduk K, Kaneko T. Chronic obstructive pulmonary disease prognostic score: A new index. Biomed Pap Med Fac Univ Palacky Olomouc Czech Repub. 2016;160(2):211-8.

2. Hu G, Wu Y, Zhou Y, Wu Z, Wei L, Li Y, et al. Prognostic role of D-dimer for in-hospital and 1-year mortality in exacerbations of COPD. Int J COPD. 2016;11(1):2729-36.

3. Hallin R, Gudmundsson G, Ulrik CS, Nieminen MM, Gislason T, Lindberg E, et al. Nutritional status and long-term mortality in hospitalised patients with chronic obstructive pulmonary disease (COPD). Respir Med. 2007;101(9):1954-60.

4. Eriksen N, Vestbo J. Management and survival of patients admitted with an exacerbation of COPD: Comparison of two Danish patient cohorts. Clin Respir J. 2010;4(4):208-14.

5. Gavazzi A, De Maria R, Manzoli L, Bocconcelli P, Di Leonardo A, Frigerio M, et al. Palliative needs for heart failure or chronic obstructive pulmonary disease: Results of a multicenter observational registry. Int J Cardiol. 2015;184:552-8.

6. Gudmundsson G, Gislason T, Lindberg E, Hallin R, Ulrik CS, Brondum E, et al. Mortality in COPD patients discharged from hospital: the role of treatment and co-morbidity. Respir Res. 2006;7:109.

7. Ho TW, Tsai YJ, Ruan SY, Huang CT, Lai F, Yu CJ. In-hospital and one-year mortality and their predictors in patients hospitalized for first-ever chronic obstructive pulmonary disease exacerbations: A nationwide population-based study. PLoS ONE. 2014;9(12).

8. García-Sanz MT, Cánive-Gómez JC, Senín-Rial L, Aboal-Viñas J, Barreiro-García A, López-Val E, et al. One-year and long-term mortality in patients hospitalized for chronic obstructive pulmonary disease. J Thorac Dis. 2017;9(3):636-45.

9. Park S, Lee SJ, Shin B, Lee SJ, Kim SH, Kwon WC, et al. The association of delta neutrophil index with the prognosis of acute exacerbation of chronic obstructive pulmonary disease. BMC Pulm Med. 2020;20(1).

10. Zimmermann M, Traxler D, Bekos C, Simader E, Mueller T, Graf A, et al. Heat shock protein 27 as a predictor of prognosis in patients admitted to hospital with acute COPD exacerbation. Cell Stress Chaperones. 2020;25(1):141-9.

11. Martinez-Rivera C, Portillo K, Muñoz-Ferrer A, Martínez-Ortiz ML, Molins E, Serra P, et al. Anemia is a mortality predictor in hospitalized patients for copd exacerbation. COPD J Chronic Obstructive Pulm Dis. 2012;9(3):243-50.

12. Stolz D, Kostikas K, Blasi F, Boersma W, Milenkovic B, Lacoma A, et al. Adrenomedullin refines mortality prediction by the BODE index in COPD: the "BODE-A" index.[Erratum appears in Eur Respir J. 2014 Dec;44(6):1718]. Eur Respir J. 2014;43(2):397-408.

13. Slenter RHJ, Sprooten RTM, Kotz D, Wesseling G, Wouters EFM, Rohde GGU. Predictors of 1-year mortality at hospital admission for acute exacerbations of chronic obstructive pulmonary disease. Respiration. 2013;85(1):15-26.

14. Bélanger M, Couillard S, Courteau J, Larivée P, Poder TG, Carrier N, et al. Eosinophil counts in first COPD hospitalizations: a comparison of health service utilization. Int J Chron Obstruct Pulmon Dis. 2018;13:3045-54.

15. Shin B, Kim SH, Yong SJ, Lee WY, Park S, Lee SJ, et al. Early readmission and mortality in acute exacerbation of chronic obstructive pulmonary disease with community-acquired pneumonia. Chronic Respir Dis. 2019;16.

16. Puhan MA, Siebeling L, Zoller M, Muggensturm P, ter Riet G. Simple functional performance tests and mortality in COPD. Eur Respir J. 2013;42(4):956-63.

17. Coleta KD, Silveira LVA, Lima DF, Rampinelli EA, Godoy I, Godoy I. Predictors of first-year survival in patients with advanced COPD treated using long-term oxygen therapy. Respir Med. 2008;102(4):512-8.

18. Neo HY, Xu HY, Wu HY, Hum A. Prediction of Poor Short-Term Prognosis and Unmet Needs in Advanced Chronic Obstructive Pulmonary Disease: Use of the Two-Minute Walking Distance Extracted from a Six-Minute Walk Test. J Palliat Med. 2017;20(8):821-8.

19. Pinto-Plata VM, Cote C, Cabral H, Taylor J, Celli BR. The 6-min walk distance: change over time and value as a predictor of survival in severe COPD. Eur Respir J. 2004;23(1):28-33.

20. Guerrero M, Crisafulli E, Liapikou A, Huerta A, Gabarrus A, Chetta A, et al. Readmission for Acute Exacerbation within 30 Days of Discharge Is Associated with a Subsequent Progressive Increase in Mortality Risk in COPD Patients: A Long-Term Observational Study. PLoS One. 2016;11(3):e0150737.

21. Ranieri P, Bianchetti A, Margiotta A, Virgillo A, Clini EM, Trabucchi M. Predictors of 6-month mortality in elderly patients with mild chronic obstructive pulmonary disease discharged from a medical ward after acute nonacidotic exacerbation. J Am Geriatr Soc. 2008;56(5):909-13.

22. Martinez FJ, Han MK, Andrei AC, Wise R, Murray S, Curtis JL, et al. Longitudinal change in the BODE index predicts mortality in severe emphysema. Am J Respir Crit Care Med. 2008;178(5):491-9.

23. Renom F, Yanez A, Garau M, Rubi M, Centeno MJ, Gorriz MT, et al. Prognosis of COPD patients requiring frequent hospitalization: role of airway infection. Respir Med. 2010;104(6):840-8.

24. Navarro A, Costa R, Rodriguez-Carballeira M, Yun S, Lapuente A, Barrera A, et al. Prognostic assessment of mortality and hospitalizations of outpatients with advanced chronic obstructive pulmonary disease. Usefulness of the CODEX index. Rev Clin Esp. 2015;215(8):431-8.

25. Niksarlioǧlu EY, Ergan Arsava B, Uǧur Demir A, Topeli Iskit A, Çöplü L. Risk factors associated with mortality of COPD patients hospitalised for exacerbation. Turk Toraks Derg. 2013;14(4):134-40.
